# A Hybrid Wetland Map for China: A Synergistic Approach Using Census and Spatially Explicit Datasets

**DOI:** 10.1371/journal.pone.0047814

**Published:** 2012-10-23

**Authors:** Kun Ma, Liangzhi You, Junguo Liu, Mingxiang Zhang

**Affiliations:** 1 School of Nature Conservation, Beijing Forestry University, Beijing, People’s Republic of China; 2 Environment and Production Technology, International Food Policy Research Institute, Washington, D.C., United States of America; 3 Ecosystems Services & Management Program, International Institute for Applied Systems Analysis, Laxenburg, Austria; Kenya Medical Research Institute - Wellcome Trust Research Programme, Kenya

## Abstract

Wetlands play important ecological, economic, and cultural roles in societies around the world. However, wetland degradation has become a serious ecological issue, raising the global sustainability concern. An accurate wetland map is essential for wetland management. Here we used a fuzzy method to create a hybrid wetland map for China through the combination of five existing wetlands datasets, including four spatially explicit wetland distribution data and one wetland census. Our results show the total wetland area is 384,864 km^2^, 4.08% of China’s national surface area. The hybrid wetland map also shows spatial distribution of wetlands with a spatial resolution of 1 km. The reliability of the map is demonstrated by comparing it with spatially explicit datasets on lakes and reservoirs. The hybrid wetland map is by far the first wetland mapping that is consistent with the statistical data at the national and provincial levels in China. It provides a benchmark map for research on wetland protection and management. The method presented here is applicable for not only wetland mapping but also for other thematic mapping in China and beyond.

## Introduction

Wetlands are the “kidney of the earth”: they play important ecological, economic, and cultural roles by providing such diverse ecosystem services as water supply, pollution control, nutrient recycling, groundwater recharge, soil formation, climate and flood regulation, and coastal protection [1], [2], [3]. Wetlands also provide recreation and tourism opportunities, and supporting vast biodiversity [4], [5]. Costanza *et al.* [6] estimated the total value of global wetland ecosystems (including coastal systems and terrestrial wetlands) to be around $15.5 trillion/yr, which is approximately 47% of the total global value of ecosystem services. However, wetlands are also a vulnerable ecosystem, and have been more seriously degraded than any other ecosystem [7], [8], [9]. During the last century, approximately 50% of the global wetlands area has been lost [10].

In China, socioeconomic development and dietary change towards meat consumption lead to increasing demand on water resources [11], [12], imposing pressures on wetland ecosystems. The main driving forces for wetland degradation include agriculture expansion, overuse of water resources and water quality deterioration, among others [13]. The Sanjiang Plain is one typical example with agriculture expansion as one major driving force as a result of the “food first” agricultural policy [14]. The Sanjiang Plain is the biggest marsh wetland in China. It is also one of the five Chinese biodiversity key areas of wetlands and fresh waters as a center of waterfowl reproduction in Northeast Asia [15]. Its marsh area decreased by 77% from 35,270 km^2^ to 8,100 km^2^ between 1954 and 2005 mainly as a result of agricultural expansion [14]. Due to the degradation of wetland, the species relying on wetland have been reducing significantly, and many rare waterfowls have been extinct [16]. The overuse of water resources within the river basin was largely responsible for the running dry downstream the Yellow river in the late 1990s [17]. Water quality deterioration is another reason for wetland degradation, for example in the Taihu lake in South China [18]. Wetland degradation is a serious ecological issue that has raised thesustainability concern. From 1990 to 2000, China’s wetlands had a net loss of 50,360 km^2^, accounting for nearly 30% of China’s natural wetland area [19], [20].

Accurate spatial information about wetlands is particularly important for national wetland protection and management. The rapid development and applications of Remote Sensing (RS), Geographical Information System (GIS) and Global Positioning System (GPS) have made wetland mapping feasible [21], [22], [23], [24]. Lehner and Döll [25] created a Global Lakes and Wetlands Database (GLWD-3) by combining a variety of existing maps, data and information. Johnston and Barson [26] mapped the location and extent of wetlands and their vegetation types using Landsat TM imagery of inland wetland sites in Victoria and New South Wales, Australia. The Canadian Wildlife Service in Quebec region tested a multi-scale object-based classification method on two sites using satellite images to map wetlands in the context of the Canadian Wetland Inventory [27]. Bowen et al. [28] utilized several geospatial data sources, including high-resolution aerial imagery, digital raster graphics, and soils data to indentify and map Playa wetlands on the High Plains, Kansas, USA. In China, between 1995 and 2003, the State Forestry Administration of the People’s Republic of China (SFA) conducted the first comprehensive census survey of the country’s wetlands covered all of China except for Taiwan, Hong Kong, and Macao. There are other recent efforts to map China’s wetlands using Landsat enhanced thematic mapper plus (ETM+) data (e.g Niu et al. 2009, Chen & Jessel 2011) [29], [30]. However, considerable disagreements on wetlands distribution exist among these maps and there are large discrepancies between the maps and national and province wetland census data [29], [31]. Despite the progress made in mapping wetland in different regions, a gridded wetland map consistent with census statistics is not yet available. This paper adopted a fuzzy agreement scoring approach [32] to create a new synergy map of China’s wetlands using five independent wetland datasets. We also presented the validation of the final synergy map.

## Methods

### 2.1 Data Sources

The following five wetlands datasets were used to create a new hybrid wetland map in this paper: Wetland-CAS, Wetland-BFU, Wetland-LU, the Global Lakes and Wetlands Database (GLWD-3), and the first wetland census in China. The formal four datasets are spatially explicit wetland distribution, while the census reports wetland areas by provinces (Table 1). We describe these datasets briefly below.

1. Wetland-CAS was produced by the Institute of Remote Sensing Applications of the Chinese Academy of Sciences (CAS) and the Beijing Normal University in China and was based mainly on Landsat Enhanced Thematic Mapper Plus (ETM+) imagery. The dataset uses manual image interpretation of satellite images, aided by elevation data, soil data, land cover/land use data, and Google Earth maps. The minimum mapping unit is equivalent to 9 ha. The Wetland-CAS map was targeted at the boundary delineation of any type of wetland except those that are under agricultural use. The total area of wetland in 2000 is 359,478 km^2^, contains three broadly classification, namely inland, coastal, and artificial wetlands. [29].
2. Wetland-BFU, created by Beijing Forestry University [33], provides the location of major wetlands listed in the first wetland census survey by SFA. Obviously, the total wetlands area from this product is much larger than the statistics from the census, but Wetland-BFU provides a good reference for the location and extent of major wetlands. The map is in a shape file format, then, we converted into a raster dataset with a spatial resolution of 1 km. The total area of wetland is 427,952 km^2^.
3. The Wetland-LU was created by the China Council for International Cooperation on Environment and Development (CCICED) by extracting different wetland types from the land-use data with spatial resolution of 1 km [30]. This dataset includes two categories of wetland: rivers and lakes, and peatland and marshes. And the total area of wetland is 227,796 km^2^.
4. The GLWD-3 was developed by Lehner and Döll [25] in a partnership between the Center for Environmental Systems Research (CESR), the University of Kassel, and the World Wildlife Fund US (WWF-US). The database was generated through the use and incorporation of data derived from proprietary products of the Environmental Systems Research Institute, Inc. (ESRI), the UNEP World Conservation Monitoring Centre (UNEPWCMC), and others. GLWD-3 is a global raster map of wetlands at a spatial resolution of 30 arc seconds, equivalent to 1 km.
5. The National Bureau of Statistics of China reports wetland census data for each province are reported by NBS [34]. The data are from the SFA’s first ever wetland census. From 1995 to 2003, the SFA did an extensive national survey of wetlands with area greater than 100 ha, including lakes, marshes, coastal wetlands, reservoirs, and rivers with their average river bed (dry channels) width greater than 10 m. The field survey investigates wetland types, area and distribution. The total area of wetland is 354,855 km^2^. This census attempted to provide more reliable data for wetland planning and management. SFA was responsible for the coordination and integration of wetland census. It allocated the census tasks to the Forestry Bureaus in each province, and also provided trainings for the provincial professionals and technicians to keep the census approach consistent. After receiving the census data from all the provinces, SFA also invited experts and scholars to evaluate the accuracy and reliability of the data. Hence, such a wetland census data is so far the most authoritative official data in China.

**Table 1:** Comparison of Wetlands Datasets.

Item	Wetland-CAS	Wetland-BFU	Wetland-LU	GLWD-3	National Wetland Census
**Year**	2000	2000	2000	1990s–2000s#	1995–2003
**Producer**	CAS*	BFU†	CCICED‡	WWF§	NBS¶
**Resolution**	1 km	Shape file	2 km	30-second	For each province
**Reference**	Niu and Gong[2009]	BFU [2011]	CCICED[2011]	Lehner and Döll [2004]	China Statistical Yearbook [2006]
**Wetlands area** **(km^2^)**	359478	427,952	227796	311000	384,855
**Wetland** **classification**	(1)River and flood plain (2)Lake (3)Marsh/swamp (4)Reservoir/pond and other man-made water bodies excluding rice field	(1)River and flood plain (2)Lake (3)Marsh, swamp, bog, fen, mire, and forest wetland (4)Coastal wetland (5)Reservoir and pond	(1)River and lake (2) Peatland and Marsh	(1)River and flood plain (2)Lake (3)Marsh, swamp, bog, fen, mire, forest/flooded forest (4)Coastal wetland (5)Reservoir	(1)River and flood plain (2)Lake (3)Marsh, swamp, bog, fen, mire, forest wetland (4)Coastal wetland (5)Reservoir and pond

Producer list:

* CAS: Chinese Academy of Science.

† BFU: Beijing Forestry University.

‡ CCICED: China Council for International Cooperation on Environment and Development.

§ WWF: World Wildlife Foundation/.

¶ NBS: National Bureau of Statistics of China.

# 1990s–2000s: GLWD-3 was produced based on various data sources, most published in the 1990s and 2000s. It is difficult to confirm which period this dataset exactly stands for.

### 2.2 Methods

#### 2.2.1 Synergy wetland map creation

**Figure 1:**
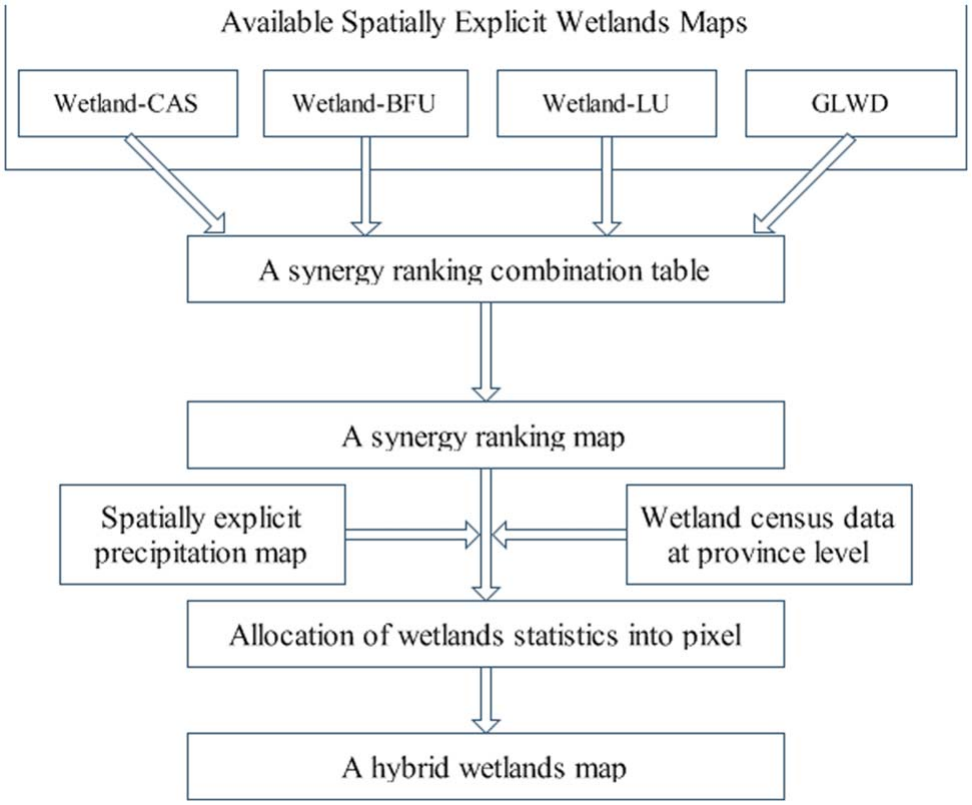
Overview of the methodology for creation of a wetland map.

All four wetland maps were first standardized into the same spatial resolution of 1 km. The basic idea behind the synergy approach is to give each pixel a score based on the agreement among different wetlands databases used in this analysis [35]. The approach allows the producer of the hybrid wetland map to rank the wetlands dataset, rather than giving all data the same weight, before they are combined. The more agreement among the maps, the higher the likelihood or probability that wetlands exist at the pixel [32]. Figure 1 illustrated the methodology in a series of steps.

The first step is to give priority order to the four wetland maps. This priority is based on three factors: authority, geographical specificity, and spatial resolution. The Wetland-CAS map, which was created based on remote sensing data, is a specific map focusing on China’s wetlands. Hence, due to its high ranking on all three priority factors, Wetland-CAS was given the highest priority. Because the Wetland-BFU map was created based on the survey wetlands data, the most authoritative set of statistics in China, we gave it the second priority. Not a specific wetland map, the Wetland-LU is based on China’s land use data with a high spatial resolution of 1 km. We therefore treated it as the third priority. The GLWD is a global map of both wetlands and lakes, which was produced based on various data sources, most published in the 1990s and 2000s. It is difficult to confirm which period this dataset exactly stands for, and so we gave it the last priority.

The second step is to create a priority rank table and give rank to each pixel. Since four different wetlands datasets were used in order of rank of confidence, there are 15 different possible combinations or confidence from 1 to 15 (Table 2). When all four wetland maps indicate a pixel as wetlands, then we give the highest rank of 1. If three maps show it is wetlands (3Y combinations), then we created the rank based on the priority orders of these three maps. In this case, for example, when GLWD (the map with the least priority) is the only map indicating no wetlands, or all other three maps with priority from 1 to 3 indicate a pixel with wetlands, we gave the highest rank for the 3Y combination (or 2, see in Table 2). But when Wetland-CAS (the map with the highest priority) is the only map indicating wetlands, we give the lowest rank for 3Y combinations (or 5, see in Table 2). A similar approach was applied for 2Y and 1Y combination. Rank 1 implies that a pixel has the highest confident of wetland existence, while rank 15 means the lowest confidence. A synergy map was then created after implementing the ranking (Fig. S1).

**Table 2:** Synergy map ranking combinations when combining four wetlands datasets.

Rank	Wetland-CAS	Wetland-BFU	Wetland-LU	GLWD
1	Y	Y	Y	Y
2	Y	Y	Y	N
3	Y	Y	N	Y
4	Y	N	Y	Y
5	N	Y	Y	Y
6	Y	Y	N	N
7	Y	N	Y	N
8	N	Y	Y	N
9	Y	N	N	Y
10	N	Y	N	Y
11	N	N	Y	Y
12	Y	N	N	N
13	N	Y	N	N
14	N	N	Y	N
15	N	N	N	Y

Here Y means the presence of wetland and N means no wetland.

The third step is to allocate statistical wetlands area to pixels. To create a raster wetland map consistent with statistics, the statistics of the wetlands area for each province must be allocated into different pixels of the synergy map. There are two situations in the allocation process. In the first situation, the total area of pixels with a rank from 1 to 15 is higher than the total wetlands area. In this case, wetlands area was first allocated to the pixels with higher rank then to the pixels with lower rank. We then added the area of pixels in a province with a rank of 1. If this area were smaller than the total wetlands area in this province, we assume all these pixels with rank 1 are wetlands. Then we add the area of pixels in this province with rank of 1 and 2. This process will repeat until the total area of pixels with a rank of 1 to certain (e.g. *i*) is larger than the statistical wetlands area. At this stage, we need to decide how to allocate wetlands in pixels with the rank of *i*. Since all of these pixels have the same rank, we used the 1960–2000 average annual precipitation data as an auxiliary criterion to reorder these pixels, and assumed the pixels with higher precipitation have a higher probability of wetlands. For example, suppose the statistical area of wetland for a province is 100 km^2^, the sum of rank from 1 to 5 is 90 km^2^, and the rank from 1 to 6 is 120 km^2^. In this case, we need to reorder the pixels of rank 6 with precipitation information and allocate the remaining 10 km^2^ of wetlands according to the criterion of precipitation.

The second situation is that the total area of pixels with a rank from 1 to 15 in a province is lower than the province’s statistical wetlands area. In this case, we assume all these pixels with ranks 1 to 15 are wetlands. We again use precipitation as an auxiliary criterion to rank the pixels and allocate the remaining wetlands area (the difference between the statistical wetlands area and the area of all pixels with ranks from 1 to 15).

#### 2.2.2 Confidence index calculation

After the hybrid wetland map being created, we calculated its confidence index. Based on the synergy map ranking in Table 2, we assign the maximum confidence, C_max_, for the highest rank (1) and the minimum confidence, C_min_, for the lowest rank (15). Knowing that there still exists some inaccuracy even for the highest rank (where all four sources agree), we assume that C_max_ is 95% and C_min_ is 5%. The confidence index between the maximum and minimum is simple linear interpolation:
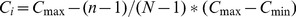
(1)


Where *C_i_* is the confidence index of pixel *i*, n represents the rank for that pixel and N is the lowest rank used for that province. N varies from province to province.

To get a general picture, we calculated overall confidence index for each province. This is a wetlands area-weighted confidence index:
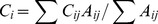
(2)


Where *C_j_* is the confidence for province *j*. *C_ij_* is the confidence for pixel *i* in province *j*. A_ij_ is the hybrid wetlands area of pixel *i* in province *j*.

#### 2.2.3 Agreement degree calculation

In order to further estimate the accuracy of the hybrid wetland, an agreement degree (AD) is used here to show the percentage of two independent datasets, the China’s Lake Dataset and the Global Reservoir and Dam Database (GRanD), captured by the different wetland maps. The AD is calculated by
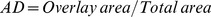
(3)


Here, the *Overlay area* is the overlay area between each wetland map and the China’s Lake Database or the GRanD within a province. The *Total area* is the total lake (or reservoir) area presented by the China’s Lake Database (or the GRanD) in the province.

## Results

### 3.1 Hybrid Wetland Map

**Figure 2:**
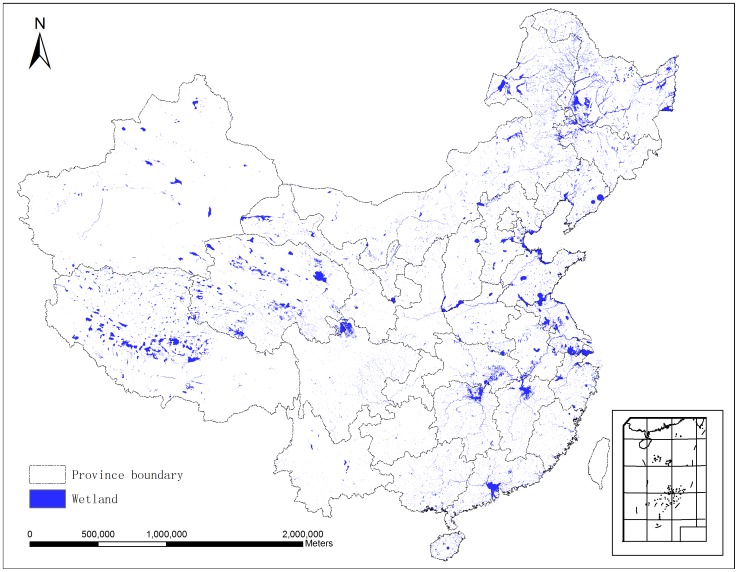
Hybrid wetland map of China. Wetland in Taiwan, Hong Kong and Macao are not included due to the lack of data.

A hybrid wetland map of China was created by applying the synergy wetland map creation methodology in each province (Fig. 2). This map shows that the total wetlands area is 384,864 km^2^, 4.08% of the total national land surface area (excluding Taiwan, Hong Kong, and Macao). The calculation compares very well with the statistical wetlands area of 384,855.26 km^2^. And the final results are very close to the wetlands area statistics in each province with a relative error range from −0.20% to 0.06% (Table S1). Tibet has the highest wetlands share, and accounts for 13.6% of the total national wetlands area. The top four provinces with the highest shares of wetland, namely Tibet, Heilongjiang, Inner Mongolia, and Qinghai, cover 46.6% of China’s total wetlands area.

The CCICED divided China into 8 ecological regions based on the scheme of ecological regionalization in China. Most wetlands in China are in the country’s Northwest and Northeast and in the Qinghai-Tibet Plateau. These three regions possess 62.1% of the wetlands in China (Fig. S2).

### 3.2 Confidence Index

**Figure 3:**
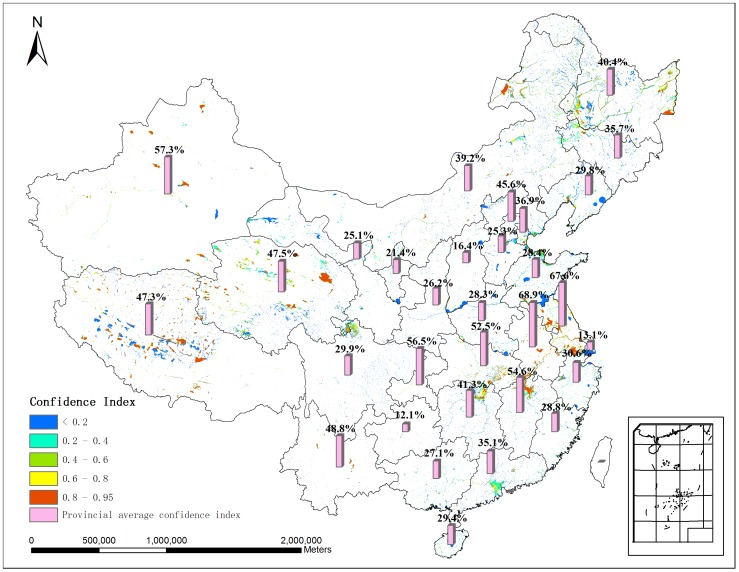
Confidence index map. The red color shows the high confidence index cells and the blue color shows the low confidence index cells. The cube refers to the average confidence index for each province.

The hybrid wetland map is created from multiple wetland maps and wetland census data. They all have inherent uncertainty which would affect the accuracy of the final hybrid wetlands distribution. Figure 3 shows the confidence index of the hybrid wetland map. About 19% of wetlands (in red) show the high confidence level with confidence index over 80%. The low confidence level (i.e. less than 20%, in blue) is widespread, covering almost 40% of wetlands area; it is in particular dominant in North China. The national average confidence index is 40.9%. There are 11 provinces with higher confidence index than the national average. Anhui is the highest province with the confidence index 68.9% (Fig. 3). While the confidence index of Guizhou province is barely over 12%, the lowest among all the 31 provinces. The provinces with low confidence index need more careful investigation in future wetlands surveys.

### 3.3 Comparison with Independent Datasets

In order to further estimate the accuracy of the hybrid wetland map, we compared the wetlands area with two independent data sets: (1) China’s Lake Database, a shape file map created by the Chinese Academy of Sciences in 2002 that shows the location and area of lakes in China; (2) The Global Reservoir and Dam Database (GRanD), which compiles reservoirs worldwide with a storage capacity of more than 0.1 km^3^.

**Figure 4:**
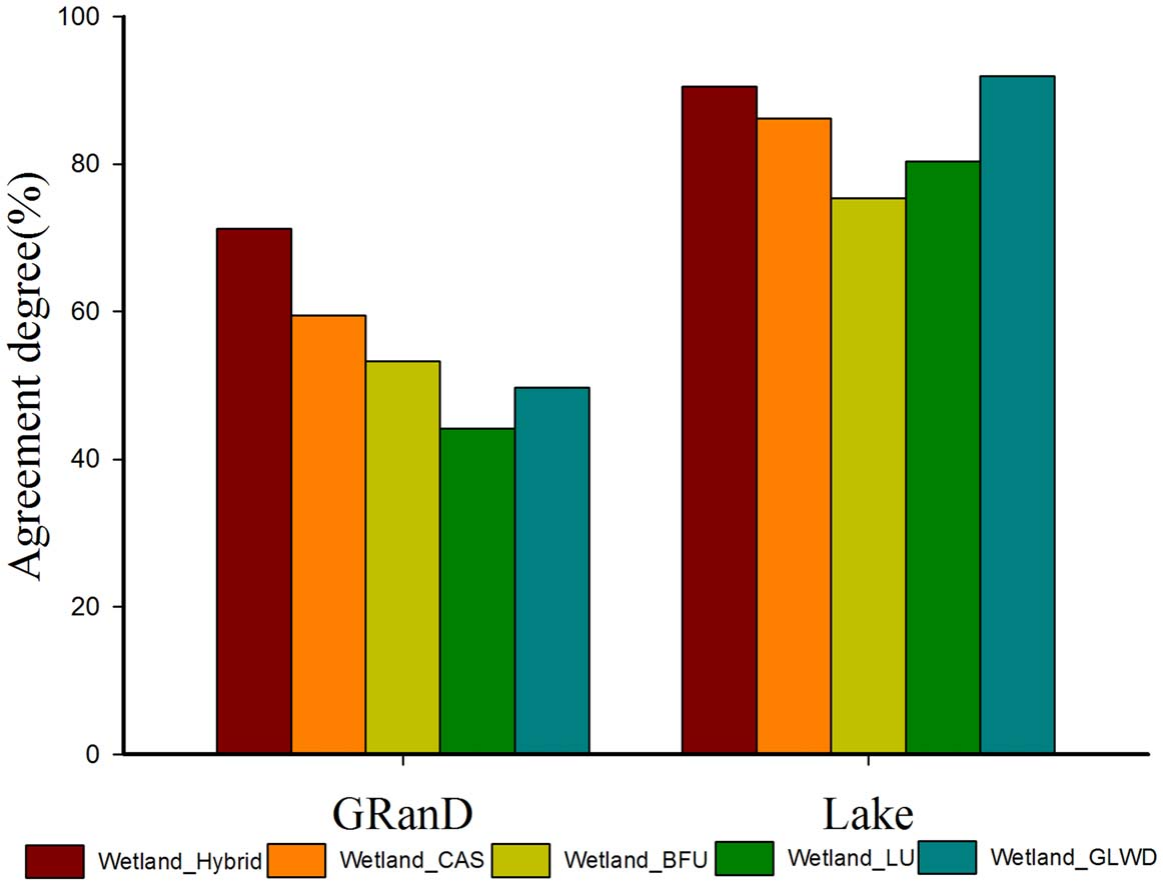
The comparison of wetlands area between the hybrid map and the existing four wetlands datasets with independent two datasets at national level. GRanD (Global Reservoir and Dam Database) and Lake (China’s Lake Database).

At the national level, for reservoirs, the AD value of the hybrid wetland map is 71.2%, which is higher than all other wetland maps. For lakes, the AD value of the hybrid wetland map is 90.5%, higher than other wetland maps except for the GLWD (Fig. 4). This is because the GLWD provides a representation of the maximum global wetlands extent [27]. At the province level, a similar pattern appears (Table S2, S3).This shows that our hybrid wetland map captures the location of most of the lakes and reservoirs in China. It is a reliable product for China’s wetland maps.

## Discussion

We synergistically combined four existing wetland maps and census data to generate a hybrid wetland map in China. This product has been published online at the Geo-wiki website (ftp://ftp.iiasa.ac.at/pub/for/Steffen/wetland_geo_wiki/), and it is available for the public for free downloading. The comparison of the hybrid wetland map with independent lake and reservoir databases shows that our hybrid map is a reliable product. This geo-referenced wetlands data provides a benchmark of wetlands in China. This hybrid wetland map is valuable for wetlands research as well as wetlands conservation and management.

When comparing the total wetland area with these four wetland maps, the synergy map is consistent with the national and provincial wetlands statistics on wetlands area, and demonstrates spatial distribution of wetlands. Considerable differences exist when comparing our map with other available spatially explicit wetland maps. One possible reason is the different degree of detail in mapping unit. For example, the total wetland area from our study is about 7% higher than the result from Wetland-CAS [29], which excluded rivers with width narrower than 90 m and wetlands with area smaller than 9 ha due to the spatial resolution constraint of remotely sensed data. But our study is based on the allocation of wetland area from the National Wetland Census by investigating wetlands with area greater than 100 ha.

Our approach used in this paper can reduce the uncertainties in mapping the national wetland. The previously available four wetland maps were given different priorities based on their reliability, geographical focus, and spatial resolution. The allocation of wetland area is based on the rank of each pixel, which was estimated by considering the priorities and agreement of the four wetland maps. This helps to allocate wetland to the pixels with higher confidence levels.

The result of confidence index mainly depends on the quality of input data. If consistent data with higher quality were available, the confidence index could be significantly improved. Due to the considerable disagreements and large discrepancy of these four datasets, the national average confidence index is only 40.9% with 20 provinces with lower values than the national average. For those provinces, our results indicate that careful wetland investigation is urgently needed to improve the accuracy of the wetland area there.

The method is elastic so that it can be easily applied when new wetlands data becomes available (e.g. the second wetlands inventory data that is coming soon). Generally, two spatially explicit datasets and one statistical data are the minimal requirements of datasets for applying this method to other regions. But we strongly suggest that at least three spatial explicit data and one accurate statistical data should be used in order to keep the hybrid map reliable.

A number of limitations in our methodology and results still remain. First, the hybrid wetland map was created from multiple wetland maps of different periods and resolution. We standardized the four previously available wetland maps into the same spatial resolution of 1 km. Some errors may occur during this process. Second, all wetland types in Ramsar Convention can be found in China, but we did not identify different wetland types in this paper; instead, we treated wetland as one type of land cover/use. Except for the wetland dataset from CCICED [30], all the other four datasets include five major wetland categories, e.g. river and flood plain, lake, marshes, coastal wetland, and reservoir/pond (see Table 1), although the sub-categories among these five main categories are to some extent different. Nevertheless, mapping different wetland types is also valuable, and we will work on this in the next step. Third, paddy crop field is an important type of wetland, or artificial wetland. Due to the lack of data in the previously available wetland maps, we did not include the paddy rice field in the current wetland product. In addition, the mapping of paddy rice field is more precise in farmland resource inventory programs than wetland census in China [29]. Last but not least, the first wetland census just surveyed the wetland with area larger than 100 hm^2^. Therefore, our current wetland area is a conservative estimate of wetland in China.

The product presented in this study is by far the first wetland mapping that is consistent with the statistical data at the national and provincial levels in China. The hybrid wetland map is valuable for wetland planning and management. The confidence level of wetland mapping can also provide insights for further wetland survey and research, e.g. more focuses should be on the regions with low values of confident indices. Applying the synergistic approach to wetland statistics in different periods can identify where and to what extent wetland is changed. Such a study is expected to be conducted when the second wetland census is available in China. The approach used in this article is applicable not only for wetland mapping but also for other thematic mapping in China and even in the rest of the world.

## Supporting Information

Figure S1 Priority rank map for China’s wetland. A rank with a small number (e.g. 1) indicates the confidence of wetlands existence is high, while a rank with a large number (e.g. 15) indicates the confidence of wetlands existence is low.
(TIF)

Figure S2 Share of wetlands area (in bracket) in different ecological regions in China.
(TIF)

Table S1 Comparison of wetlands area between the provincial statistics and aggregated provincial wetlands area from the hybrid wetland map.
(DOCX)

Table S2 Agreement degree of five geo-referenced wetland maps (including the hybrid wetland map Hybrid from this study) with China’s Lake Database for each province.
(DOCX)

Table S3 Agreement degree of five geo-referenced wetland maps (including the hybrid wetland map Hybrid from this study) with Global Reservoir and Dam Database for each province.
(DOCX)

## References

[1] Woodward RT, Wui YS (2001) The economic value of wetland services: a meta-analysis. Ecological Economics 37: 257–270.

[2] Leibowitz SG (2003) Isolated wetlands and their functions: an ecological perspective. Wetlands 23: 517–531.

[3] Millennium EA (2005) Ecosystems and Human Well-being: Wetlands and Water Synthesis.

[4] Jiang M, Lu XG, Xu LS, Chu LJ, Tong SZ (2007) Flood mitigation benefit of wetland soil–A case study in Momoge National Nature Reserve in China. Ecological Economics 61: 217–223.

[5] Adhikari AR, Acharya K, Shanahan SA, Zhou XP (2011) Removal of nutrients and metals by constructed and naturally created wetlands in the Las Vegas Valley, Nevada. Environmental Monitoring and Assessment 180: 97–113. 21125423 10.1007/s10661-010-1775-y

[6] Costanza R, d’Arge R, Groot Rd, Farber S, Grasso M, et al (1997) The value of the world’s ecosystem services and natural capital. nature 387: 253–260.

[7] Turner RK, van den Bergh JCJM, Söderqvist T, Barendregt A, van der Straaten J, et al (2000) Ecological-economic analysis of wetlands: scientific integration for management and policy. Ecological Economics 35: 7–23.

[8] Vörösmarty CJ, McIntyre PB, Gessner MO, Dudgeon D, Prusevich A, et al (2010) Global threats to human water security and river biodiversity. nature 467: 555–561. 20882010 10.1038/nature09440

[9] Carpenter SR, Stanley EH, Zanden MJV (2011) State of the World’s Freshwater Ecosystems: Physical, Chemical, and Biological Changes. Annual Review of Environment and Resources 36: null.

[10] Zedler JB, Kercher S (2005) WETLAND RESOURCES: Status, Trends, Ecosystem Services, and Restorability. Annual Review of Environment and Resources 30: 39–74.

[11] Liu JG, Savenije HHG (2008) Food consumption patterns and their effect on water requirement in China. Hydrology and Earth System Science. 12: 887–898.

[12] Liu JG, Yang H, Savenije HHG (2008) China’s move to higher-meat diet hits water security. Nature. 454: 397. 10.1038/454397a 18650891

[13] Yang Z, Chen B (2011) Systematic studies on wetlands in China. Ecological Modelling 222: 221–223.

[14] Wang Z, Song K, Ma W, Ren C, Zhang B, et al. (2011) Loss and Fragmentation of Marshes in the Sanjiang Plain, Northeast China, 1954–2005. Wetlands: 1–10.

[15] Chen LZ (1998) Biodiversity: Current situation and policy of conservation in China. Beijing: Science Press. 197–204.

[16] Liu HY, Lu XG, Liu ZQ (2000) Landscape planning and ecology construction of wetland comprehensive protected area system in the Sanjiang Plain. Journal of Environmental Sciences. 12: 361–366.

[17] Zhang JF, Sun QX (2005) Causes of wetland degradation and ecological restoration in the Yellow River Delta Region. Forestry Studies in China 7: 15–18.

[18] Qu CS, Chen W, Bi J, Huang L, Li FY (2011) Ecological risk assessment of pesticide residues in Taihu Lake wetland, China. Ecological Modelling 222: 287–292.

[19] Cyranoski D (2009) Putting China’s wetlands on the map. nature 458: 134. 19279595 10.1038/458134a

[20] Gong P, Niu ZG, Cheng X, Zhao KY, Zhou DM, et al (2010) China’s wetland change (1990–2000) determined by remote sensing. SCIENCE CHINA Earth Sciences 53: 1036–1042.

[21] Chopra R, Verma V, Sharma P (2001) Mapping, monitoring and conservation of Harike wetland ecosystem, Punjab, India, through remote sensing. International Journal of Remote Sensing 22: 89–98.

[22] Prigent C, Matthews E, Aires F, Rossow WB (2001) Remote sensing of global wetland dynamics with multiple satellite data sets. Geophys Res Lett 28: 4631–4634.

[23] Ozesmi SL, Bauer ME (2002) Satellite remote sensing of wetlands. Wetlands Ecology and Management 10: 381–402.

[24] Rebelo LM, Finlayson C, Nagabhatla N (2009) Remote sensing and GIS for wetland inventory, mapping and change analysis. Journal of Environmental Management 90: 2144–2153. 18367311 10.1016/j.jenvman.2007.06.027

[25] Lehner B, Döll P (2004) Development and validation of a global database of lakes, reservoirs and wetlands. Journal of Hydrology 296: 1–22.

[26] Johnston RM, Barson MM (1993) Remote sensing of Australian wetlands: An evaluation of Landsat TM data for inventory and classification. Marine and Freshwater Research 44: 235–252.

[27] Grenier M, Demers AM, Labrecque S, Benoit M, Fournier RA, et al (2007) An object-based method to map wetland using RADARSAT-1 and Landsat ETM images: test case on two sites in Quebec, Canada. Canadian Journal of Remote Sensing 33: S28–S45.

[28] Bowen MW, Johnson WC, Egbert SL, Klopfenstein ST (2010) A GIS-based approach to identify and map playa wetlands on the High Plains, Kansas, USA. Wetlands 30: 675–684.

[29] Niu ZG, Gong P, Cheng X, Guo JH, Wang L, et al (2009) Geographical characteristics of China’s wetlands derived from remotely sensed data. Science in China Series D: Earth Sciences 52: 723–738.

[30] Chen YY, Jessel B (2011) Ecosystem services and management strategy in China. Beijing: China Environmental Science Press.

[31] Yang B, Wang Y, Zhu D (1997) The Tidal Flat Resource of China. Journal of Natural Resources 12: 307–316.

[32] Fritz S, You L, Bun A, See L, McCallum I, et al (2011) Cropland for sub-Saharan Africa: A synergistic approach using five land cover data sets. Geophysical Research Letters 38: L04404.

[33] BFU (2011) Mapping China’s Wetland based on the first National Wetland Census. Beijing Forestry University.

[34] National Bureau of Statistics of China (2006) China Statistical Yearbook (2006). Beijing: China Statistics Press.

[35] Jung M, Henkel K, Herold M, Churkina G (2006) Exploiting synergies of global land cover products for carbon cycle modeling. Remote Sensing of Environment 101: 534–553.

