# Supplementary material for: A Hybrid Wetland Map for China: A Synergistic Approach Using Census and Spatially Explicit Datasets
Source: PLoS One. 2012 Oct 23;7(10):e47814. doi: 10.1371/journal.pone.0047814 (PMC3479119; doi:10.1371/journal.pone.0047814)
Supplement: Table S1 — Comparison of wetlands area between the provincial statistics and aggregated provincial wetlands area from the hybrid wetland map. (DOCX) [file pone.0047814.s003.docx]

**Table S1** Comparison of wetlands area between the provincial statistics and aggregated provincial wetlands area from the hybrid wetland map

| Province | *S* (km^2^) | *H* (km^2^) | *RE* |
| --- | --- | --- | --- |
| Anhui | 6538.7 | 6538 | 0.01% |
| Beijing | 343.59 | 344 | -0.12% |
| Chongqing | 432.06 | 432 | 0.01% |
| Fujian | 4429.97 | 4429 | 0.02% |
| Gansu | 12580.96 | 12606 | -0.20% |
| Guangdong | 13980.74 | 13981 | 0.00% |
| Guangxi | 6561.07 | 6561 | 0.00% |
| Guizhou | 794.1 | 794 | 0.01% |
| Hainan | 3114.69 | 3115 | -0.01% |
| Hebei | 10819.39 | 10820 | -0.01% |
| Heilongjiang | 43148.35 | 43143 | 0.01% |
| Henan | 6241.29 | 6241 | 0.00% |
| Hubei | 9273.29 | 9273 | 0.00% |
| Hunan | 12269.09 | 12269 | 0.00% |
| Jiangsu | 16746.7 | 16746 | 0.00% |
| Jiangxi | 9987.97 | 9988 | 0.00% |
| Jilin | 12033.56 | 12034 | 0.00% |
| Liaoning | 12196.15 | 12202 | -0.05% |
| Neimenggu | 42450.48 | 42450 | 0.00% |
| Ningxia | 2556.43 | 2555 | 0.06% |
| Qinghai | 41259.67 | 41257 | 0.01% |
| Shandong | 17840.99 | 17837 | 0.02% |
| Shanghai | 3197.14 | 3197 | 0.00% |
| Shaanxi | 2928.95 | 2928 | 0.03% |
| Shanxi | 4999 | 5000 | -0.02% |
| Sichuan | 9616.8 | 9617 | 0.00% |
| Tianjin | 1717.8 | 1719 | -0.07% |
| Xinjiang | 14101.56 | 14101 | 0.00% |
| Xizang | 52320 | 52312 | 0.02% |
| Yunnan | 2353.05 | 2354 | -0.04% |
| Zhejiang | 8021.72 | 8021 | 0.01% |
| Total | 384855.26 | 384864 | 0.00% |

In this table, *S* is the statistic of wetlands area of each province from national wetland census data, *H* is the aggregated wetlands area of province *i* from the hybrid wetland map. The relative error (*RE*) is calculate as

RE = (S - H) / S * 100%
