# Supplementary material for: A Hybrid Wetland Map for China: A Synergistic Approach Using Census and Spatially Explicit Datasets
Source: PLoS One. 2012 Oct 23;7(10):e47814. doi: 10.1371/journal.pone.0047814 (PMC3479119; doi:10.1371/journal.pone.0047814)
Supplement: Table S2 — Agreement degree of five geo-referenced wetland maps (including the hybrid wetland map Hybrid from this study) with China’s Lake Database for each province. (DOCX) [file pone.0047814.s004.docx]

**Table S2** Agreement degree of five geo-referenced wetland maps (including the hybrid wetland map *Hybrid* from this study) with China’s Lake Database for each province

| Province | *Hybrid* | *Wetland-CAS* | *Wetland-BFU* | *Wetland-LU* | *GLWD* |
| --- | --- | --- | --- | --- | --- |
| Anhui | 93.3% | 89.1% | 88.4% | 82.2% | 89.2% |
| Beijing | 89.1% | 82.0% | 74.9% | 56.3% | 73.8% |
| Chongqing | NA* | NA | NA | NA | NA |
| Fujian | NA | NA | NA | NA | NA |
| Gansu | 100.0% | 100.0% | 0.0% | 100.0% | 100.0% |
| Guangdong | NA | NA | NA | NA | NA |
| Guangxi | NA | NA | NA | NA | NA |
| Guizhou | 89.5% | 89.5% | 26.3% | 36.8% | 89.5% |
| Hainan | NA | NA | NA | NA | NA |
| Hebei | 99.5% | 75.9% | 92.0% | 85.6% | 74.3% |
| Heilongjiang | 92.6% | 88.9% | 81.5% | 71.5% | 72.9% |
| Henan | NA | NA | NA | NA | NA |
| Hubei | 82.4% | 80.5% | 48.1% | 69.9% | 92.1% |
| Hunan | 93.6% | 86.5% | 59.1% | 76.0% | 88.8% |
| Jiangsu | 96.0% | 90.6% | 99.8% | 88.9% | 89.8% |
| Jiangxi | 88.8% | 84.1% | 68.9% | 70.8% | 93.1% |
| Jilin | 92.4% | 82.5% | 75.4% | 60.9% | 75.5% |
| Liaoning | 92.9% | 82.1% | 85.7% | 82.1% | 89.3% |
| Neimenggu | 90.0% | 79.9% | 65.1% | 75.5% | 80.3% |
| Ningxia | 85.7% | 52.4% | 47.6% | 57.1% | 38.1% |
| Qinghai | 97.3% | 94.4% | 88.0% | 87.9% | 94.2% |
| Shandong | 99.4% | 76.0% | 92.5% | 93.3% | 86.0% |
| Shanghai | 100.0% | 75.4% | 100.0% | 84.1% | 98.6% |
| Shaanxi | 78.7% | 78.7% | 39.3% | 59.0% | 4.9% |
| Shanxi | 100.0% | 91.7% | 100.0% | 33.3% | 75.0% |
| Sichuan | 72.2% | 78.6% | 60.3% | 41.3% | 39.7% |
| Tianjin | 59.7% | 28.2% | 46.8% | 75.8% | 1.6% |
| Xinjiang | 94.1% | 92.8% | 72.3% | 87.5% | 94.8% |
| Xizang | 85.1% | 82.2% | 68.1% | 78.3% | 97.5% |
| Yunnan | 95.8% | 89.7% | 88.3% | 77.9% | 94.2% |
| Zhejiang | 95.0% | 73.3% | 76.7% | 60.0% | 73.3% |

Here NA means there is no lake data in the provinces with China’s Lake Database
