# Supplementary material for: A Hybrid Wetland Map for China: A Synergistic Approach Using Census and Spatially Explicit Datasets
Source: PLoS One. 2012 Oct 23;7(10):e47814. doi: 10.1371/journal.pone.0047814 (PMC3479119; doi:10.1371/journal.pone.0047814)
Supplement: Table S3 — Agreement degree of five geo-referenced wetland maps (including the hybrid wetland map Hybrid from this study) with Global Reservoir and Dam Database for each province. (DOCX) [file pone.0047814.s005.docx]

**Table S3** Agreement degree of five geo-referenced wetland maps (including the hybrid wetland map *Hybrid* from this study) with Global Reservoir and Dam Database for each province

| Province | *Hybrid* | *Wetland-CAS* | *Wetland-BFU* | *Wetland-LU* | *GLWD* |
| --- | --- | --- | --- | --- | --- |
| Anhui | 57.3% | 49.1% | 62.5% | 34.9% | 55.8% |
| Beijing | 92.8% | 84.9% | 79.6% | 65.8% | 77.0% |
| Chongqing | 32.4% | 43.5% | 38.5% | 12.2% | 40.2% |
| Fujian | 55.5% | 37.6% | 0.0% | 20.4% | 20.4% |
| Gansu | 80.5% | 67.5% | 67.5% | 47.4% | 64.9% |
| Guangdong | 70.2% | 57.9% | 40.0% | 42.8% | 51.1% |
| Guangxi | 66.3% | 40.7% | 34.6% | 29.8% | 33.4% |
| Guizhou | 51.9% | 50.6% | 0.0% | 21.2% | 37.8% |
| Hainan | 89.3% | 77.3% | 76.7% | 14.0% | 32.0% |
| Hebei | 86.2% | 58.4% | 47.0% | 53.5% | 44.2% |
| Heilongjiang | 75.0% | 75.0% | 0.0% | 0.0% | 0.0% |
| Henan | 85.4% | 68.7% | 66.8% | 51.2% | 55.6% |
| Hubei | 45.6% | 49.9% | 39.2% | 35.2% | 41.3% |
| Hunan | 65.6% | 47.7% | 31.2% | 21.1% | 30.9% |
| Jiangsu | 96.4% | 93.8% | 99.5% | 91.4% | 94.9% |
| Jiangxi | 59.0% | 51.7% | 34.4% | 33.5% | 38.4% |
| Jilin | 76.9% | 59.1% | 51.7% | 41.8% | 69.0% |
| Liaoning | 74.8% | 59.6% | 57.1% | 42.8% | 46.5% |
| Neimenggu | 73.1% | 62.1% | 49.5% | 47.3% | 51.1% |
| Ningxia | 91.9% | 14.1% | 50.5% | 11.1% | 52.5% |
| Qinghai | 83.5% | 81.7% | 81.1% | 76.4% | 1.5% |
| Shandong | 81.2% | 52.5% | 22.7% | 40.9% | 40.9% |
| Shanghai | NA* | NA | NA | NA | NA |
| Shaanxi | 71.0% | 40.0% | 54.7% | 29.8% | 34.3% |
| Shanxi | 91.4% | 53.6% | 44.6% | 53.6% | 45.5% |
| Sichuan | 40.8% | 36.4% | 36.6% | 13.5% | 2.8% |
| Tianjin | 86.0% | 71.0% | 58.0% | 83.0% | 34.0% |
| Xinjiang | 0.0% | 0.0% | 0.0% | 0.0% | 0.0% |
| Xizang | 98.2% | 86.4% | 90.7% | 74.6% | 95.6% |
| Yunnan | 23.1% | 20.4% | 19.7% | 12.9% | 20.4% |
| Zhejiang | 81.6% | 57.5% | 58.7% | 43.0% | 50.3% |

Here NA means there is no reservoir data in the provinces with Global Reservoir and Dam Database
